# Yearly fluctuations of flower landscape in a Mediterranean scrubland: Consequences for floral resource availability

**DOI:** 10.1371/journal.pone.0191268

**Published:** 2018-01-18

**Authors:** Víctor Flo, Jordi Bosch, Xavier Arnan, Clara Primante, Ana M. Martín González, Helena Barril-Graells, Anselm Rodrigo

**Affiliations:** 1 CREAF, Center for Ecological Research and Forestry Applications, Campus UAB, Bellaterra, Spain; 2 Center for Macroecology, Evolution and Climate, Natural History Museum of Denmark, University of Copenhagen, Universitetsparken, Copenhagen Ø, Denmark; 3 Ecology Unit, Autonomous University of Barcelona, Bellaterra, Spain; Indiana University Bloomington, UNITED STATES

## Abstract

Species flower production and flowering phenology vary from year to year due to extrinsic factors. Inter-annual variability in flowering patterns may have important consequences for attractiveness to pollinators, and ultimately, plant reproductive output. To understand the consequences of flowering pattern variability, a community approach is necessary because pollinator flower choice is highly dependent on flower context. Our objectives were: 1) To quantify yearly variability in flower density and phenology; 2) To evaluate whether changes in flowering patterns result in significant changes in pollen/nectar composition. We monitored weekly flowering patterns in a Mediterranean scrubland community (23 species) over 8 years. Floral resource availability was estimated based on field measures of pollen and nectar production per flower. We analysed inter-annual variation in flowering phenology (duration and date of peak bloom) and flower production, and inter-annual and monthly variability in flower, pollen and nectar species composition. We also investigated potential phylogenetic effects on inter-annual variability of flowering patterns. We found dramatic variation in yearly flower production both at the species and community levels. There was also substantial variation in flowering phenology. Importantly, yearly fluctuations were far from synchronous across species, and resulted in significant changes in floral resources availability and composition at the community level. Changes were especially pronounced late in the season, at a time when flowers are scarce and pollinator visitation rates are particularly high. We discuss the consequences of our findings for pollinator visitation and plant reproductive success in the current scenario of climate change.

## Introduction

Flower production and flowering phenology are species-specific traits that are strongly constrained by life form and phylogeny [1,2]. However, these traits are also influenced by extrinsic factors such as weather variables [3–7] and nutrient availability [8–10], and therefore show a certain level of variation across years [11–13]. Inter-annual variability in flowering patterns may have important consequences in terms of attractiveness to pollinators and flower predators, and ultimately affect plant reproductive output. For example, large floral displays (number of flowers per individual) (reviewed in [14]) and high flower densities [15–18] usually result in increased pollinator visitation rates. Yearly variability in flowering patterns may also have a strong impact on the reproductive success of flower-visiting insects. In situations in which a given species does not bloom profusely, flower visitors foraging on this plant take longer to gather food resources [19,20], and may be forced to switch to alternative, non-preferred flower species [21].

Interest in yearly variability in flowering time has increased in the current scenario of climate change, especially in relation to potential temporal mismatches between plants and their pollinators [7,22–27]. On the other hand, much less attention has been given to variation in flower production [12,13,22,28,29]. Most studies have focused on individual species and studies at the community level remain scarce. A community approach is essential to fully understand the consequences of variation in flowering patterns for interacting species such as pollinators, because flower choice by pollinators is highly dependent on floral context (abundance and composition of co-flowering species) [18,21,30,31]. If yearly changes in flowering patterns are pronounced and, most importantly, if they are not synchronous across species in the community, flower-visiting insects may encounter a different "flower landscape" from year to year. Such temporal variability could have important consequences for the structure of plant-pollinator interactions and pollination function. Alternatively, if changes in flowering patterns are small and/or all species in the community fluctuate in parallel, flower composition and interactions with pollinators may remain relatively consistent through time, a scenario that would favour specialization in plant-pollinator interactions [32].

In this study, we monitored flower production and flowering phenology in a coastal scrubland community (23 species) over 8 years. Our specific objectives are: 1) To quantify yearly variability in flowering patterns in terms of flower density and phenology. We are interested in establishing whether the various species in the community fluctuate synchronously or not. Our flower community is strongly seasonal [33,34]. For this reason, we are also interested in potential interactions between inter-annual and seasonal (intra-annual) variability; 2) To evaluate to what extent changes in flowering patterns result in significant changes in flower resource (pollen and nectar) availability and composition.

## Methods

### Study site

The study area is a Mediterranean scrubland located in the Garraf Natural Park, near Barcelona, NE Spain. Field work was conducted with permission of the Park’s administration. The study plot (~ 1ha; UTM: 409345.0, 4569737.5) is located 340 m above sea level and 1700 m from the coastline. The vegetation is dominated by *Quercus coccifera*, *Pistacia lentiscus*, *Thymus vulgaris* and *Rosmarinus officinalis*. The climate is characterized by warm dry summers and mild winters. Most precipitation occurs in autumn and spring. Mean annual temperature is 15.5°C and yearly rainfall is around 600 mm.

### Flower counts

Data collection took place from March to June of years 2006 to 2014 (except 2010). Bloom becomes extremely scarce in July due to the severe summer drought. We used 6 permanent 50 x 1 m transects forming a grid. Distance between adjacent parallel transects was 20 m. Once a week, all open flowers of entomophilous species were counted in these transects. Flower life span was less than a week in all species. We analyze the data corresponding to the 23 most abundant species, representing 15 plant families, and amounting to 99.4% of the total number of flowers counted throughout the study.

We characterized the flowering pattern of each species based on the total number of flowers produced per hectare, year and transect (flowering density), the duration (in weeks) of the flowering period (flowering duration) and the date (week; week 1 = first week of March) of maximum flowering intensity (flowering peak). We do not analyze date of flowering onset because it was strongly correlated to flowering peak date every year (Pearson’s correlation, n = 23 species, r = 0.67–0.91; all p < 0.001), in addition, peak is a better measure for phenological comparisons [35]. Although in some years *Rosmarinus officinalis* started its bloom earlier than our flower-sampling campaign, we always captured most of the flowering period. Since sampling dates were not exactly the same each year, we use linear interpolations to work with the same days of the year (DOY) across the eight years. Nine species did not bloom at all in at least one of the eight years. For these species, flowering peak and flowering duration values are calculated excluding years of null flowering.

### Nectar and pollen production

To understand whether fluctuations in flowering pattern resulted in temporal changes in floral resource composition we measured pollen (mm^3^) and nectar production per flower (mg of sugar in 24 h) in the 23 plant species. These measurements were taken in 2006 and 2007, depending on the species. On haphazardly chosen plants, we used nylon bags to enclose branches with mature buds. After 24 h we extracted and measured the nectar accumulated in individual flowers by using Drummond micropipettes of 0.25, 0.50 and 1 μl (samples size = 18–144 flowers per species; S1 Table). To measure flower pollen content, we selected between 10 and 15 flower buds per species. Flower buds were removed and kept in vials with 70% ethanol and taken to the laboratory. Each flower bud was dissected under a stereomicroscope and the number of anthers was counted. Then, three anthers per flower were removed, suspended in 2 ml of 70% ethanol and sonicated in a water bath for 2–4 minutes to dislodge pollen grains and 9 ml of isotonic solution was added. The number of pollen grains in the resulting suspension was then estimated using an electronic particle counter (Coulter Multisizer) with 200 μm aperture. From these data we obtained the total number of pollen grains produced per flower. The use of data from two years for the entire eight-year series assumes that intra-specific pollen and nectar production per flower was consistent throughout the duration of the study. Both pollen and nectar production per flower are known to vary at the species level from year to year [36–38]. However, because in our study inter-specific differences in pollen and nectar production per flower are as high as 276-fold and 178-fold, respectively (see Results), we assume that the relatively much smaller intra-specific yearly differences will not significantly alter our community level results. Flower density data and pollen/nectar production per flower were combined to obtain estimates of pollen and nectar production per ha.

### Data analysis

#### Variability in flowering patterns

We used Linear Mixed-effect Models (LMMs) to analyze the effect of year, species and the interaction between year and species on flowering density, flowering peak and flowering duration. A significant year effect and lack of significant year x species interaction would indicate that all species in the community fluctuate more or less in synchrony. Transect was added as a random factor because the same transects were repeatedly sampled over the years. Eight species did not bloom at all in at least one of the eight years. For these species/years, flower density was 0 and flowering peak and flower duration could not be calculated. Consequently, these species were removed from the analyses. Flowering density was log-transformed and residuals were checked to satisfy normality and homoscedasticity assumptions. Analyses were performed with the *lmer* function of lme4 package [39] in R (version 3.3.3, R Core Team, Vienna (Austria), 2017, https://www.R-project.org).

To analyze yearly and seasonal variability in overall (sum of all species) flower density, we conducted a LMM with flower density as the dependent variable and year and month (March, April, May, June) as factors. The month x year interaction was also included in the model. Transect was added as a random factor. The same analysis was subsequently repeated with nectar and pollen production data. Flower, nectar and pollen data were log-transformed to achieve normality. Nectar and pollen results were very similar. For this reason, only nectar results are shown.

To analyse yearly and seasonal variation in flower composition we conducted permutational multivariate analyses of variance (PERMANOVA). The response variable was a Bray-Curtis dissimilarity matrix of flower composition (flower density of each species) between the different sampling events (combinations of years and months). Year, month and their interaction were included as predictors, and transects were selected as groups (*strata*) within which permutations were restricted. The analysis was executed with 9999 permutations, using *Adonis* function of *vegan* package [40] in R. To facilitate interpretation of the results, non-metric multidimensional scaling (NMDS) was performed based on flower composition Bray-Curtis distances using the *metaMDS* function of *vegan* package in R. Subsequently, equivalent PERMANOVA and NMDS analyses were conducted with nectar and pollen composition data. Again, because pollen and nectar results were very similar, we only show nectar results. All analyses were conducted without 10 species that had missing values in some transects during the entire season for the 8 years of sampling.

#### Phylogenetic constraints

To find out whether inter-annual flowering pattern variability was constrained by phylogeny, we conducted tests of phylogenetic signal for continuous variables based on Bloomberg’s K assuming Brownian motion character evolution [41]. We first built a phylogenetic tree of the 23 species with *phylomatic V*.*3* [42]. Polytomies were resolved manually using *timetree* databases [43]. Then, we used the *phylosig* function of *phytools* R package [44] to detect phylogenetic signal in flowering density CV, flowering peak SD, and flowering duration SD. To estimate the significance of the observed phylogenetic signals, K values obtained with the real data were compared to K values obtained from randomizations (1000 trees in which species where shuffled across the tips of the phylogenetic tree) [41]. We found that variability in flower density, flowering peak and flowering duration was not constrained by phylogeny (S2 Table). That is, phylogenetically related species did not show similar propensity to variation (or lack thereof) in flowering patterns. Therefore, phylogeny was not accounted for in subsequent analyses.

## Results

### Variation in flowering phenology and density and consequences for floral resource availability

**Fig 1:**
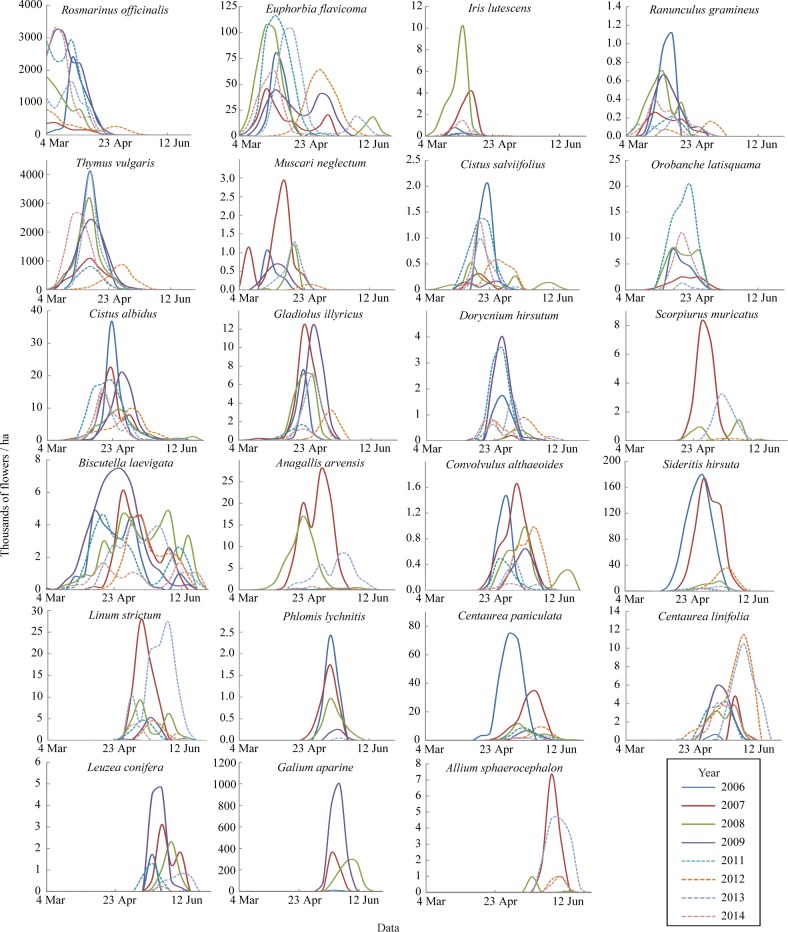
Yearly flowering curves of the 23 most abundant plant species of the Garraf community. Ordered by timing of peak bloom. Note different scales on y-axis.

Yearly variation in flower density was strongly species-dependent (Fig 1). Some species (e.g., *Cistus albidus*, *Euphorbia flavicoma*) had relatively consistent flower density, while others (e.g., *Scorpiurus muricatus*, *Galium aparine*) fluctuated dramatically (flowering density CV > 1.6; S3 Table). Maximum differences (ratio between highest and lowest years) were 267-fold in *Leuzea conifera* and 135-fold in *Linum strictum* (Fig 1), and eight species did not bloom at all in one or more years. As for flowering phenology, some species showed important shifts in flowering peak date (e.g., *Anagallis arvensis*, *Cistus salviifolius*) while others were much more consistent (e.g., *Linum strictum*, *Orobanche latisquama*) (S3 Table, Fig 1). Different species also showed different levels of variation in flowering duration. For example, *Scorpiurus muricatus* and *Iris lutescens* were highly variable, while *Euphorbia flavicoma* and *Biscutella laevigata* were relatively consistent (S3 Table, Fig 1). In all species, the CV of flower density was higher than the CV of flowering duration (S3 Table). Differences across years in flower density, flowering peak and flowering duration of the 23 species were highly significant (Table 1). Importantly, the interaction year x species was also highly significant for the three variables (Table 1), indicating that yearly variation in flowering patterns was strongly asynchronous across species.

**Table 1:** Anova tables of the fitted LMMs analyzing yearly changes in mean flower density (log-transformed), flowering peak, and flowering duration of the 15 most abundant species of the Garraf community.

Variable	Factor	DF	F	P
Flower density	Year	7	4.6	< 0.001
	Species	14	197.0	< 0.001
	Year x Species	98	2.8	< 0.001
Flowering peak	Year	7	31.9	< 0.001
	Species	14	243.0	< 0.001
	Year x Species	98	4.1	< 0.001
Flowering duration	Year	7	20.5	< 0.001
	Species	14	72.0	< 0.001
	Year x Species	98	3.3	< 0.001

**Fig 2:**
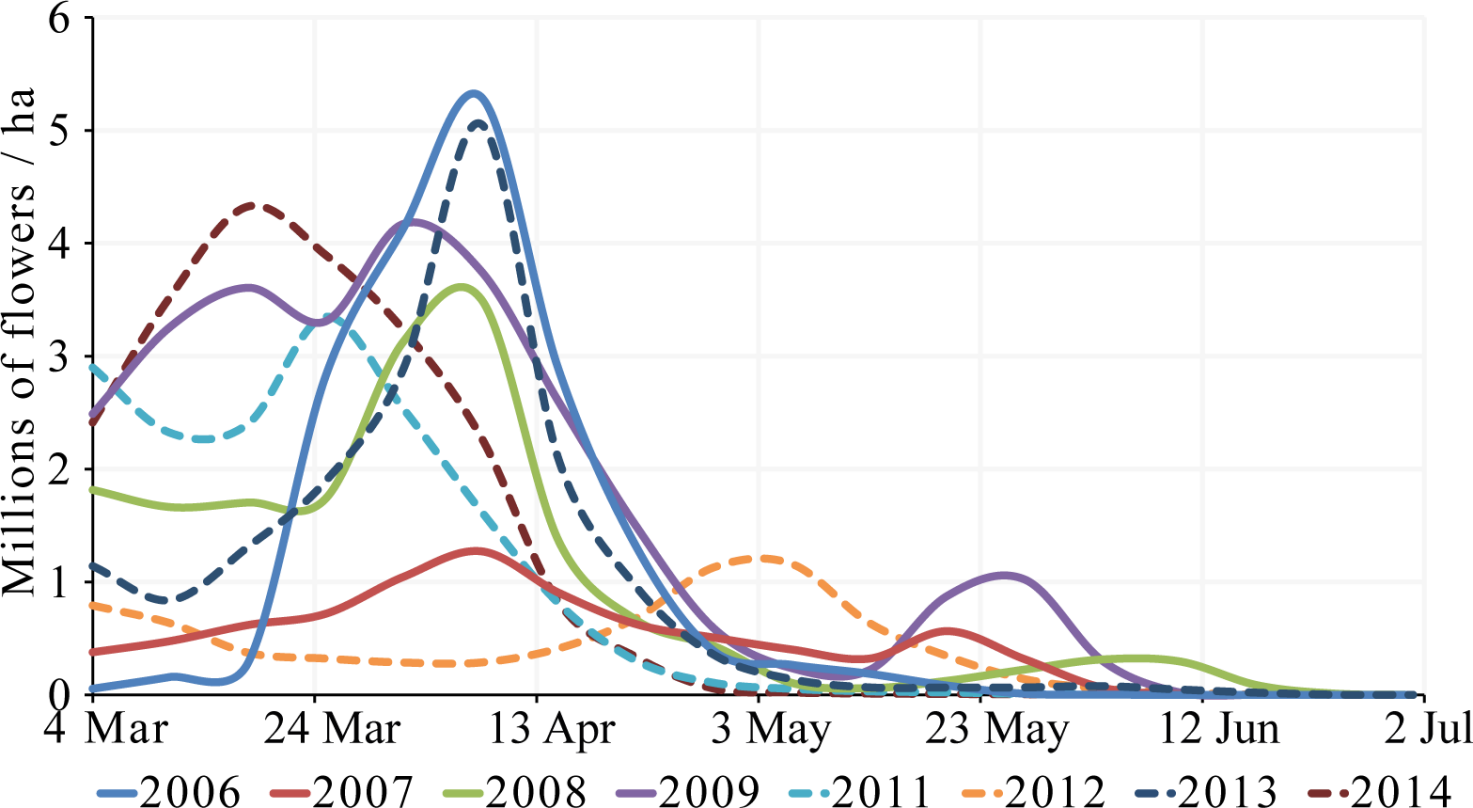
Yearly overall (23 species) flowering curves of the Garraf community. Each curve represents the mean value across transects (n = 6).

The community was largely dominated by two species, *Rosmarinus officinalis* and *Thymus vulgaris*, which together accounted for 91.7 ± 6.5% of the total (8-year) flower density. Because these two species showed important maximum yearly differences in flower density (10- and 4-fold, respectively), overall flower availability was highly variable across years (Fig 2, Table 2). In most years, total flower production ranged from 16 to 21 million flowers / ha (Fig 2). However, in 2007 and 2012 flower production was only _~_ 8 million flowers / ha, and in 2009 it was _~_ 28 million flowers / ha. The seasonal distribution of flower density also changed noticeably across years. In most years, flowering peaked in late March or early April, but in 2014 peak bloom occurred in mid-March, and in 2012 in early May (Fig 2).

**Table 2:** Anova tables of the fitted LMMs analyzing yearly and monthly variation in overall (23 species) flower density and nectar availability in the Garraf community.

Variable	Factor	DF	F	P
Flower density (flowers / ha)	Year	7	11.9	< 0.001
	Month	3	235.7	< 0.001
	Year x Month	21	10.5	< 0.001
Nectar availability (sugar mg / ha)	Year	7	17.9	< 0.001
	Month	3	756.4	< 0.001
	Year x Month	21	17.6	< 0.001

**Fig 3:**
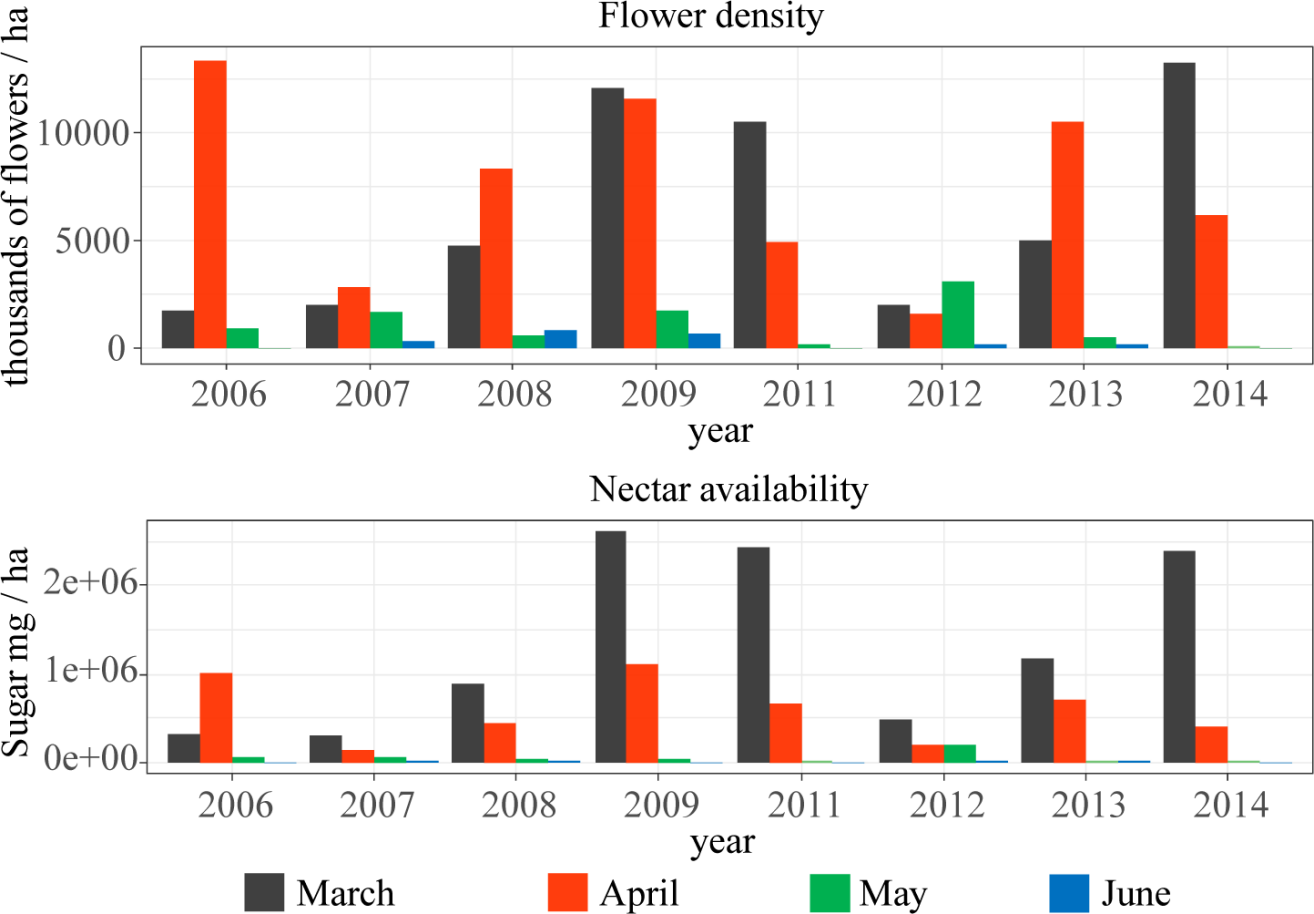
Monthly distribution of flower density and nectar availability in the Garraf community across eight years.

As expected given the strong seasonal component of the flower community, overall flower density also varied dramatically across months (Table 2). Of more interest is the highly significant year x month interaction, indicating that annual variation is far from uniform across seasons (Table 2, Fig 3). Importantly, changes in overall flower density had consequences for nectar availability, which also presented high inter- and intra-annual variability (Fig 3), and a highly significant year x month interaction (Table 2). Nectar availability was usually highest in March, but in 2006 maximum availability occurred in April (Fig 3).

### Variation in flower composition and consequences for floral resource composition

**Fig 4:**
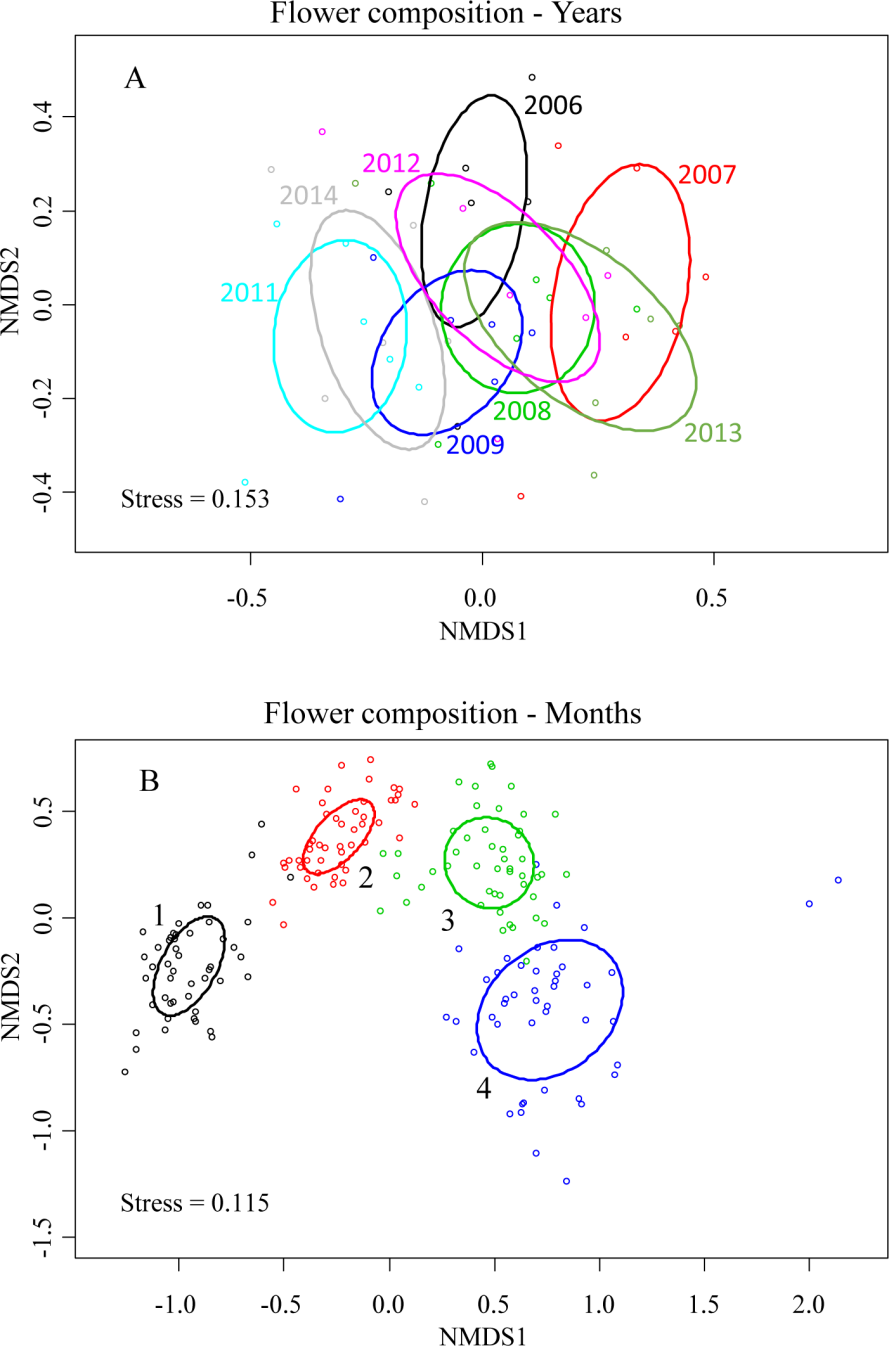
Non-metric multidimensional scaling (NMDS) analysis describing yearly and monthly variation in flower composition. Ellipses correspond to standard deviations of the sampling events of each grouping factor (years or months). 1: March; 2: April; 3: May; 4: June. Points on figure (A) represents transect–year values. Points on figure (B) represents each transect–month–year combination. The results for nectar composition were similar and are shown in S1 Fig.

Flower composition differed significantly across years (Table 3, Fig 4A), although the factor year explained only a small part of the observed variability (9.2%). As mentioned, the Garraf flower community is largely dominated by two species, *Rosmarinus officinalis* and *Thymus vulgaris* that bloom profusely in early spring. Such dominance is likely to mask changes in flower composition involving non-dominant species. As expected, flower composition differed strongly across months (Table 3, Fig 4B), explaining 32.6% of the observed variability. More importantly, the year x month interaction was also strongly significant (Table 3), indicating that the expected pattern of seasonal variability was not consistent across years. Yearly variability in flower composition was greatest at the end of the flowering period (June; Fig 4B). Variability in nectar composition closely paralleled the results obtained with flower composition (Table 3). Interestingly, consecutive years often had contrasting nectar compositions (e.g., 2006 *vs*. 2007; 2013 *vs*. 2014).

**Table 3:** Results of PERMANOVAs analyzing yearly and monthly variation in flower and nectar composition in the Garraf community.

Flower composition	DF	F	R^2^	P
Year	7	5.84	0.09	< 0.001
Month	3	48.27	0.33	< 0.001
Year x Month	21	4.70	0.22	< 0.001
Residuals	160		0.36	
Total	191		1	
Nectar composition				
Year	7	6.73	0.085	< 0.001
Month	3	72.32	0.394	< 0.001
Year x Month	21	6.05	0.230	< 0.001
Residuals	160		0.290	
Total	191		1	

## Discussion

Our study demonstrates that flowering patterns change dramatically from year to year both at the species and community levels. At the species level, most variability is due to changes in flower density, rather than flowering phenology. Some studies have addressed the influence of environmental factors on flowering patterns (see [13] for a novel methodological approach). In general, these studies have shown flower production to be positively related to rainfall [4,22]. On the other hand, flowering phenology appears to be mostly regulated by temperature, with warmer temperatures advancing flowering periods [7,45–47].

Importantly, our study also demonstrates that yearly flowering fluctuations are by no means synchronized across species, resulting in significant yearly changes in flower composition, especially late in the season (June). Flower seasonal composition is important because most pollinator species in Garraf have short activity periods in relation to the overall flowering period of the community, and the the Garraf pollination network is structured in seasonal modules [33,48]. By June, flower density in the Garraf scrubland dramatically declines, and visitation rates (visits per flower and time unit) are very high [33,34]. At that time, it is not infrequent to see several pollinators foraging simultaneously on the same inflorescence. Two species blooming in June, *Sideritis hirsuta* and *Galium aparine* show marked fluctuations in flower density (Fig 1), resulting in drastic yearly changes in flower resource availability. Thus, June-active pollinators are exposed to floral resources that are both scarce and unreliable from year to year, a situation that is expected to hinder foraging specialization [32].

Changes in flower density and composition are known to affect pollinator flower choice and visitation rates, with potentially important consequences for plant reproductive success [16,49–51]. Changes in flower visitation rates have been shown to affect stigma pollen deposition, sometimes resulting in changes in seed-set [52,53]. Plant reproductive success is also likely to be affected by changes in pollinator composition. Different pollinator species differ in their pollinating efficiency both in terms of number of pollen grains delivered per visit [54–57], and the quality of the pollen deposited (e.g. levels of geitonogamy [54, 58,59]). Changes in flower composition may also influence indirect interactions among plant species competing for pollinators or facilitating pollinator visitation [15,60–63]. Because the effects of flower neighborhood on pollinator visitation are density-dependent (facilitation may turn into competition as the facilitating species becomes increasingly abundant) [15,63], shifts in flower composition may change the direction of these interactions.

Changes in pollen and nectar availability imply that pollinators are confronted with inconsistent floral resource landscapes from year to year, with potential ecological consequences for their fitness. In years with low flower densities, pollinators are forced to fly longer distances to gather pollen/nectar loads. These increased foraging costs result in slow nest provisioning rates and decreased offspring body size [19,64,65], ultimately leading to increased developmental and wintering mortality [19,66]. Long provisioning trips are also likely to result in increased parasitism by cleptoparasites and parasitoids that enter bee nests and lay their eggs while the nest founder is away foraging [67,68].

In the current scenario of climate change, the Mediterranean Basin is predicted to experience important temperature increases [69,70]. This is expected to advance the flowering time of most plant species [7,45–47]. Pollinators may be able to track these phenological changes if, as suggested by current evidence [26,71–74], they also respond to global warming by advancing their activity period. We know less about the potential effects of climate change on flower production. Increased temperature has been shown to have either positive or negative effects on flower production depending on the species [75]. However, the predicted increase in the occurrence of drought episodes in the Mediterranean Basin [69,70] is expected to increase the frequency of years with low floral resources [4,22]. Our study shows shifts in flowering intensity to be as least as important as shifts in flowering time, and to be highly asynchronous across species. For this reason, and given the irregular occurrence of drought episodes, we expect shifts in flowering density to have high negative effects on pollinators.

## Supporting information

S1 Table Mean nectar and pollen production per flower. (PDF)

S2 Table Results of analyses exploring phylogenetic (Bloomberg’s K test) constraints on flowering pattern variability. (PDF)

S3 Table Descriptive statistics of flower density, flowering peak and flowering duration of the 23 main plant species of the Garraf community. Species ordered by timing of flowering peak.
(PDF)

S1 Fig Non-metric multidimensional scaling (NMDS) analysis describing yearly and monthly variation in nectar composition. Ellipses correspond to standard deviations of the sampling events of each grouping factor (years or months). 1: March; 2: April; 3: May; 4: June. Points on figure (A) represents transect–year values. Points on figure (B) represents each transect–month–year combination.
(PDF)

S1 Dataset Database used in the analyses. (ZIP)
