# Supplementary material for: Yearly fluctuations of flower landscape in a Mediterranean scrubland: Consequences for floral resource availability
Source: PLoS One. 2018 Jan 18;13(1):e0191268. doi: 10.1371/journal.pone.0191268 (PMC5773194; doi:10.1371/journal.pone.0191268)
Supplement: S1 Table — (PDF) [file pone.0191268.s001.pdf]

S1 Table. Mean and standard deviation of nectar and pollen production per flower of the 23 plant species studied.

Specie	Nectar (sugar mg / flower)			Pollen (mm^3 / flower)		
	Mean	SD	N	Mean	SD	N
Alium sphaerocephalon	0.081	0.062	19	3.603	1.002	10
Anagallis arvensis	0.000	0.000	*	0.072	0.019	10
Biscutella laevigata	0.017	0.020	34	0.117	0.031	15
Centaurea linifolia	0.064	0.052	29	0.209	0.052	11
Centaurea paniculata	0.022	0.034	144	0.126	0.041	10
Cistus albidus	0.205	0.120	34	6.747	3.574	15
Cistus salvifolius	0.045	0.039	37	5.796	1.387	10
Convolvulus altheoides	0.066	0.064	22	2.145	0.498	10
Dorycnium hirsutum	0.079	0.062	27	0.224	0.096	10
Euphorbia flavicoma	0.011	0.023	121	0.336	0.108	10
Gallium lucidum	0.000	0.000	*	0.102	0.016	10
Gladiolus illyricus	0.224	0.190	18	2.185	0.965	10
Iris lutescens	0.000	0.000	0	8.981	5.308	10
Leuzea conifera	0.036	0.027	61	0.033	0.013	10
Linum strictum	0.003	0.003	21	0.298	0.041	10
Muscari neglectum	0.036	0.020	39	2.388	0.553	14
Orobanche latisquama	0.260	0.244	30	0.829	0.278	10
Phlomis lychnitis	0.535	0.259	32	0.495	0.134	10
Ranunculus gramineus	0.000	0.000	*	8.891	3.710	10
Rosmarinus officinalis	0.243	0.148	84	0.912	0.321	10
Scorpiurus muricatus	0.011	0.014	37	0.577	0.109	10
Siderites hirsuta	0.044	0.040	50	0.057	0.020	15
Thymus vulgaris	0.023	0.018	64	0.160**	0.124	10

* No nectar production

** Hermaphroditic flowers (pollen production in female flowers = 0).
