# Supplementary material for: Yearly fluctuations of flower landscape in a Mediterranean scrubland: Consequences for floral resource availability
Source: PLoS One. 2018 Jan 18;13(1):e0191268. doi: 10.1371/journal.pone.0191268 (PMC5773194; doi:10.1371/journal.pone.0191268)
Supplement: S2 Table — (PDF) [file pone.0191268.s002.pdf]

S2 Table. Results of analyses exploring phylogenetic (Bloomberg's K test) constraints on flowering pattern variability.

	K	P
Variation (CV) in flower density	0.53	0.280
Variation (SD) in flowering peak	0.66	0.124
Variation (SD) in flowering duration	0.40	0.682
