# Supplementary material for: Yearly fluctuations of flower landscape in a Mediterranean scrubland: Consequences for floral resource availability
Source: PLoS One. 2018 Jan 18;13(1):e0191268. doi: 10.1371/journal.pone.0191268 (PMC5773194; doi:10.1371/journal.pone.0191268)
Supplement: S3 Table — Species ordered by timing of flowering peak. (PDF) [file pone.0191268.s003.pdf]

S3 Table. **Descriptive statistics of flower density, flowering peak and flowering duration of the 23 main plant species of the Garraf community.** Species ordered by timing of flowering peak.

		Flower density (flowers/ha)			Flowering peak (week)		Flowering duration (weeks)		
Species	Family	Mean	SD	CV	Mean	SD	Mean	SD	CV
Rosmarinus officinalis	Lamiaceae	8328720	5247094	0.63	3	1.31	13	3.11	0.24
Thymus vulgaris	Lamiaceae	7351342	3234590	0.44	6	2.00	13	2.90	0.22
Euphorbia flavicoma	Euphorbiaceae	294603	106057	0.36	6	1.51	14	1.83	0.13
Ranunculus gramineus	Ranunculaceae	1443	736	0.51	6	1.96	7	1.96	0.28
Iris lutescens	Iridaceae	4318	6693	1.55	6	1.67	5	2.80	0.56
Muscari neglectum	Liliaceae	2098	2581	1.23	6	1.75	5	2.07	0.41
Cistus albidus	Cistaceae	54460	15249	0.28	8	1.07	13	3.48	0.27
Cistus salvifolius	Cistaceae	2300	1311	0.57	8	2.07	7	2.12	0.30
Orobanche latisquama	Orobanchaceae	19949	22143	1.11	8	1.04	6	3.11	0.52
Gladiolus illyricus	Iridaceae	15543	10414	0.67	9	1.06	7	1.41	0.20
Biscutela laevigata	Brassicaceae	27560	10197	0.37	10	1.06	14	2.07	0.15
Dorycnium hirsutum	Fabaceae	4114	3209	0.78	10	2.00	6	2.97	0.50
Anagallis arvensis	Primulaceae	24886	36831	1.48	10	3.14	8	2.49	0.31
Scorpiurus muricatus	Fabaceae	4045	6796	1.68	10	2.12	5	3.63	0.73
Sideritis hirsuta	Lamiaceae	186707	280061	1.50	11	1.31	9	1.75	0.19
Phlomis lychnitis	Lamiaceae	1376	1830	1.33	11	1.39	4	2.77	0.69
Convolvulus althaeoides	Convolvulaceae	2377	1569	0.66	11	1.49	7	2.33	0.33
Linum strictum	Linaceae	30259	35100	1.16	11	0.55	7	1.64	0.23
Centaurea linifolia	Asteraceae	18370	13226	0.72	12	1.07	7	1.07	0.15
Centaurea paniculata	Asteraceae	58697	75719	1.29	12	1.30	8	2.76	0.35
Leuzea conifera	Asteraceae	3541	3399	0.96	13	1.19	4	1.41	0.35
Allium sphaerocephalon	Amaryllidaceae	4011	6057	1.51	14	1.63	5	1.86	0.37
Galium aparine	Rubiaceae	472212	783872	1.66	14	1.22	4	1.52	0.38
