# Supplementary material for: Yearly fluctuations of flower landscape in a Mediterranean scrubland: Consequences for floral resource availability
Source: PLoS One. 2018 Jan 18;13(1):e0191268. doi: 10.1371/journal.pone.0191268 (PMC5773194; doi:10.1371/journal.pone.0191268)
Supplement: S1 Fig — Ellipses correspond to standard deviations of the sampling events of each grouping factor (years or months). 1: March; 2: April; 3: May; 4: June. Points on figure (A) represents transect–year values. Points on figure (B) represents each transect–month–year combination. (PDF) [file pone.0191268.s004.pdf]

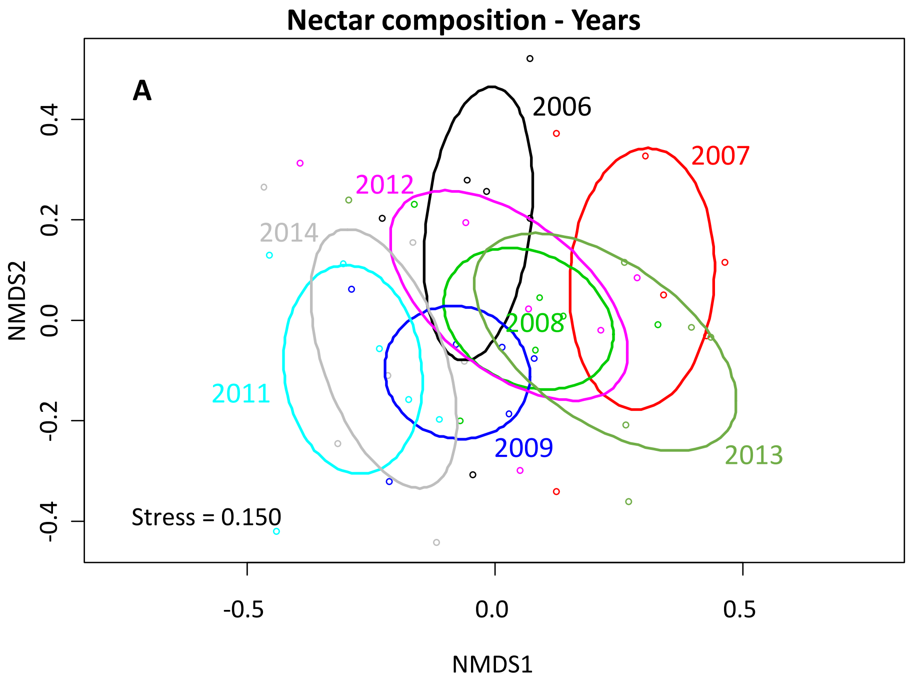

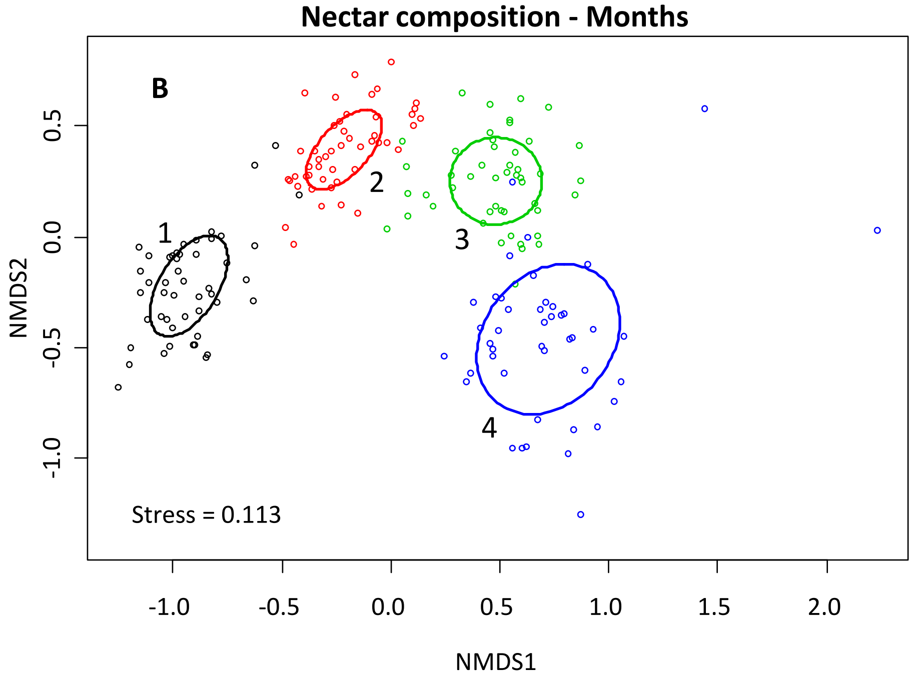

S1 Fig. Non-metric multidimensional scaling (NMDS) analysis describing yearly and monthly variation in nectar composition. Ellipses correspond to standard deviations of the sampling events of each grouping factor (years or months). 1: March; 2: April; 3: May; 4: June. Points on figure A represents transect – year values. Points on figure B represents each transect – month – year combination.
